# A Prospective Study of the Operative Treatment of Clavicular Fractures

**DOI:** 10.7759/cureus.102481

**Published:** 2026-01-28

**Authors:** Ranj Bhakar, Arjun S Chakrapani, Arfaz Shaik, Aaron Alexander, Thivagar Murugesan, Prasanna Kumar Anbazhagan, Dan Ghent

**Affiliations:** 1 Department of Trauma and Orthopaedics, Torbay and South Devon NHS Trust, Torquay, GBR; 2 Department of Orthopaedics, Croydon University Hospital, London, GBR; 3 Trauma and Orthopaedics, Wrightington, Wigan and Leigh NHS Foundation Trust, Wigan, GBR; 4 Department of Orthopaedics, University Hospital Llandough, Penarth, GBR; 5 Department of Orthopaedic Surgery, Shrewsbury and Telford NHS Foundation Trust, Shrewsbury, GBR; 6 Department of Upper Gastrointestinal Surgery, Ashford and St Peter's Hospitals NHS Foundation Trust, Chertsey, GBR; 7 Core Medical Training, Queens Medical Centre, Nottingham, GBR

**Keywords:** clavicular fracture, functional outcome, locking compression plate, operative fixation, prospective study

## Abstract

Objective

This study aimed to evaluate the functional and radiological outcomes associated with the operative management of displaced midshaft clavicular fractures using pre-contoured locking compression plates (LCPs), specifically in cases that met surgical indications such as fracture displacement, shortening, or comminution.

Methodology

In this prospective study, 30 adult patients with displaced midshaft or lateral clavicular fractures underwent open reduction and internal fixation (ORIF) with pre-contoured LCPs at a tertiary care center. Patients were followed clinically for six months postoperatively. Functional outcomes were assessed using the Constant-Murley Score (CMS), and radiological union was evaluated through serial radiographs. Data were analyzed using SPSS Statistics version 25.0 (IBM Corp., Armonk, NY), and results were reported as mean ± standard deviation (SD).

Results

The mean age of the patients was 36.4 ± 10.2 years, with 21 patients (70%) being male. The mean time to radiographic union was 12.8 ± 2.3 weeks. At the final follow-up, the mean Constant-Murley Score (CMS) was 91.2 ± 6.4, with 24 patients (80%) demonstrating excellent shoulder function. Complications were limited to two patients (6.7%), while three patients (10%) experienced superficial infections or implant-related irritation. There were no observed cases of nonunion or implant failure.

Conclusions

Operative fixation of displaced clavicular fractures using pre-contoured LCPs provides stable fixation, facilitates early mobilization, and results in excellent functional recovery with minimal complications. Surgical management may be considered in active adults with displaced fractures to help optimize outcomes.

## Introduction

Clavicle fractures are among the most common skeletal injuries, representing approximately 2.6-4% of all adult fractures and 35-45% of shoulder girdle injuries [1]. The clavicle acts as a strut between the axial and appendicular skeletons, helping to preserve shoulder alignment and enable upper limb mobility. Fractures most commonly occur in the midshaft region, which is the thinnest portion of the bone with relatively limited muscular and ligamentous support [2]. Typical mechanisms of injury include road traffic accidents, falls onto the outstretched hand, and direct trauma to the shoulder. Nonoperative management typically results in healing through secondary bone union and is generally effective for minimally displaced fractures. However, displaced or comminuted fractures are associated with a higher risk of malunion, shortening, and nonunion, which may result in cosmetic deformity, persistent pain, and reduced shoulder strength [3,4]. Nonunion rates of up to 15% have been reported in displaced midshaft fractures managed conservatively

With advances in fracture biomechanics and rising functional demands, operative fixation has become increasingly common. Surgical management aims to achieve anatomical reduction, restore the original clavicular length, and facilitate early mobilization to maximize shoulder function [6]. Pre-contoured locking compression plates (LCPs) offer improved biomechanical stability, minimize hardware-related complications, and allow early return to routine activities, particularly in young and active individuals [7]. Despite these advantages, the indications for surgery remain a matter of debate. Potential risks include infection, implant irritation, and hardware prominence, although several studies have demonstrated superior functional outcomes compared with conservative treatment [8,9]. Determining patient subgroups most likely to benefit from surgical intervention remains an important clinical consideration.

Recent randomized controlled trials (RCTs), including those by the Canadian Orthopaedic Trauma Society, demonstrate that plate fixation of displaced midshaft clavicular fractures results in faster union, lower nonunion rates, and better early functional outcomes compared with conservative management [10]. However, long-term superiority and complication profiles across diverse populations remain unclear. Moreover, few prospective studies from developing countries, including India, have systematically evaluated functional and radiological outcomes following fixation with modern pre-contoured locking plates.

In this context, the present prospective study was undertaken to evaluate clinical outcomes of operative management of displaced clavicular fractures in adults at a tertiary care center. Specifically, the study aimed to assess radiological union following open reduction and internal fixation (ORIF) with pre-contoured LCPs, evaluate functional outcomes using the Constant-Murley Score (CMS), and document postoperative complications. This study provides valuable evidence regarding the efficacy, functional recovery, and safety of operative fixation of clavicular fractures in the Indian clinical setting [11]. This study aimed to evaluate the functional and radiological outcomes of operative management of displaced midshaft clavicular fractures using pre-contoured LCPs, specifically in cases meeting surgical indications such as fracture displacement, shortening, or comminution.

## Materials and methods

Study design and setting

This prospective observational study was conducted over one year, from 2016 to 2017, in the Department of Orthopaedics at Chhindwara Institute of Medical Sciences, Chhindwara, Madhya Pradesh, a tertiary care teaching hospital. Ethical approval was obtained from the Institutional Ethics Committee (reference number: CIMS/IRB/PG/2016/187), and all procedures were performed in accordance with the Declaration of Helsinki. Written informed consent was obtained from all participants before enrollment. Patients were recruited using consecutive sampling, whereby all eligible patients presenting during the study period were included until the required sample size was achieved. This approach minimized selection bias and enhanced the external validity of the findings.

Outcome measures

The primary outcome of the study was functional recovery of the affected shoulder at six months postoperatively, assessed using the Constant-Murley Score [12], a validated scoring system that evaluates pain, activities of daily living, range of motion, and muscle strength, providing a comprehensive measure of shoulder function. Secondary outcomes included time to radiological union, assessed by serial postoperative radiographs, and the incidence of postoperative complications, including superficial or deep infection, implant failure, nonunion, and hardware irritation. Additional secondary measures comprised the shoulder range of motion and overall clinical recovery documented during scheduled follow-up visits.

Sample size calculation

The sample size was calculated based on an expected mean CMS of 85 with a standard deviation (SD) of 10, as reported in previous studies evaluating surgical fixation of displaced clavicular fractures [9,13]. The standard formula for estimating a population mean was applied:

\[ n = \left( \frac{Z_{\alpha/2} \times \sigma}{d} \right)^2 \]

\ where Z_{\alpha/2} = 1.96 corresponds to a 95\% confidence level, \sigma = 10 is the standard deviation, and d = 5 represents the desired absolute precision. Substituting the values yields:

\[ n = \left( \frac{1.96 \times 10}{5} \right)^2 = (3.92)^2 = 15.37 \] The minimum required sample size was rounded up to 16 patients. After accounting for an anticipated attrition rate of 20\%, the final target sample size was 33 patients.

Selection criteria

Patients aged 18 years or older presenting with acute clavicular fractures suitable for operative fixation were included. The study specifically comprised displaced midshaft or lateral-third fractures of less than two weeks’ duration, classified according to the Robinson classification system [14] as type 2B1 (displaced simple or wedge fractures) or type 2B2 (displaced comminuted fractures), as these fracture patterns are associated with poorer outcomes following nonoperative management. Patients with open or pathological fractures, associated neurovascular injury, or polytrauma involving the ipsilateral upper limb or shoulder girdle were excluded, as these conditions could independently influence functional and radiological outcomes. Those who were unwilling or unable to comply with the prescribed postoperative follow-up schedule were also excluded to ensure completeness and reliability of outcome assessment.

Preoperative assessment and surgical technique

All patients underwent a standardized preoperative evaluation that included detailed history taking, clinical examination, and documentation of baseline demographic and injury-related variables, including age, sex, side of injury, mechanism of injury, and time from injury to surgery. Radiological evaluation consisted of standard anteroposterior and 45° cephalic tilt views of the clavicle, with fractures classified according to the Robinson system.

Surgical procedures were performed by experienced orthopedic surgeons under general anesthesia or interscalene block, with patients positioned supine and appropriately supported. Open reduction and internal fixation were performed through a superior approach using a pre-contoured 3.5-mm locking compression plate. Fracture reduction and implant placement were performed according to established operative principles, with intraoperative fluoroscopic confirmation of reduction and implant positioning. Wound closure and postoperative immobilization were performed according to standard institutional protocols [15,16].

Postoperative management and rehabilitation

Postoperative care was standardized for all patients. Prophylactic intravenous antibiotics were administered at anesthetic induction and continued for 24 hours in accordance with the guidelines [17]. Closed suction drains, when used, were removed within 24-48 hours based on output. Analgesia followed a multimodal regimen, including paracetamol and nonsteroidal anti-inflammatory drugs, with opioids reserved for breakthrough pain.

Early rehabilitation was initiated on the first postoperative day with passive and pendulum shoulder exercises, followed by active-assisted and active range-of-motion exercises at two to three weeks, depending on pain tolerance and clinical assessment. Strengthening exercises were introduced after radiological evidence of fracture union. Patients were reviewed at two weeks, six weeks, three months, and six months postoperatively for clinical and radiological evaluation, following standardized follow-up protocols [15,16]. Radiological outcomes were independently assessed by two consultant orthopedic surgeons blinded to clinical outcomes. Union was defined as bridging callus across at least three cortices on two orthogonal radiographic views. Functional outcomes were assessed using the Constant-Murley Score by a single independent assessor who was blinded to radiological findings, minimizing observer and assessment bias.

Statistical analysis

Data were entered into Microsoft Excel and analyzed using IBM SPSS Statistics version 25.0 (IBM Corp., Armonk, NY). Quantitative variables, including age, time to union, and CMS, were summarized as mean ± standard deviation (SD). Categorical variables, such as sex, fracture laterality, and complications, were presented as frequencies and percentages.

## Results

Table 1 presents the demographic characteristics of the 30 enrolled patients. The study population consisted of 21 (70.0%) males and nine (30.0%) females. The mean age of the cohort was 36.4 ± 10.2 years (range: 19-58 years). This reflects a predominance of young to middle-aged males, which aligns with their greater exposure to outdoor activities and road-related injuries.

**Table 1:** Demographic characteristics of the study population SD: standard deviation

Parameter	Category	Frequency (n=30)	Percentage (%)
Gender	Male	21	70.0
	Female	9	30.0
Age (years)	Mean ± SD	36.4 ± 10.2	-
Age range (years)	19–58	-	-

Table 2 summarizes the mechanisms of injury. Road traffic accidents were the most common cause, accounting for 18 (60.0%) patients, followed by falls onto an outstretched hand in nine (30.0%) patients and direct shoulder trauma in three (10.0%) patients. These findings indicate that high-energy mechanisms were responsible for the majority of displaced clavicular fractures.

**Table 2:** Distribution of cases according to mode of injury

Mode of injury	Number of patients	Percentage (%)
Road traffic accident	18	60.0
Fall on outstretched hand	9	30.0
Direct trauma	3	10.0

Table 3 outlines the fracture distribution. Midshaft fractures were identified in 25 (83.3%) patients, whereas lateral-third fractures occurred in five (16.7%) patients. Regarding laterality, 19 (63.3%) fractures involved the right side and 11 (36.7%) involved the left. This confirms that midshaft injuries remain the most common subtype requiring surgical fixation.

**Table 3:** Distribution of fractures by anatomical site and laterality

Fracture type	Number of patients	Percentage (%)
Midshaft	25	83.3
Lateral third	5	16.7
Side involved	Right: 19	63.3
	Left: 11	36.7

Radiological outcomes are shown in Table 4. All patients achieved fracture union, with a mean time to radiological union of 12.8 ± 2.3 weeks (range: 10-17 weeks). Notably, 24 (80.0%) patients demonstrated satisfactory callus formation by the 12-week follow-up. No cases of delayed union or nonunion were observed.

**Table 4:** Radiological union time SD: standard deviation

Parameter	Mean ± SD (weeks)	Range (weeks)
Time to radiological union	12.8 ± 2.3	10–17

Table 5 reports functional outcomes at six months using the Constant-Murley Score. Excellent results were achieved in 24 (80.0%) patients, good results in five (16.7%) patients, and fair results in one (3.3%) patient. The mean Constant-Murley Score was 91.2 ± 6.4, indicating excellent shoulder function in the majority of participants.

**Table 5:** Functional outcome (Constant–Murley Shoulder Score) at six months SD: standard deviation

Functional grade	Score range	Number of patients	Percentage (%)
Excellent	≥90	24	80.0
Good	80–89	5	16.7
Fair	<80	1	3.3
Mean ± SD	91.2 ± 6.4	-	-

Postoperative complications are summarized in Table 6. Superficial wound infection occurred in two (6.7%) patients, while hardware irritation was reported in three (10.0%) patients. No cases of deep infection, implant failure, or nonunion were recorded. The overall complication rate was 16.7% (five patients), reflecting a low incidence of postoperative adverse events.

**Table 6:** Postoperative complications

Complication	Number of patients	Percentage (%)
Superficial infection	2	6.7
Hardware irritation	3	10.0
Nonunion	0	0.0
Implant failure	0	0.0

## Discussion

Clavicular fractures are among the most common injuries of the shoulder girdle, accounting for 2.6-5% of all adult fractures and 35-45% of shoulder girdle injuries. Historically, displaced midshaft fractures were managed conservatively, often with acceptable outcomes. However, recent evidence have highlighted the limitations of nonoperative treatment, including higher rates of nonunion, malunion, and persistent shoulder dysfunction. Consequently, operative fixation has gained favor to achieve anatomical reduction, enable early mobilization, and improve functional outcomes, particularly for displaced or comminuted fractures [15].

We evaluated 30 adult patients who underwent ORIF for displaced midshaft and lateral-third clavicular fractures using pre-contoured locking compression plates. Our results were consistent with previous literature, showing excellent union rates, favorable functional outcomes, and a low incidence of complications. The mean age of the cohort was 36.4 ± 10.2 years, and 70% were male, consistent with the findings of Shen et al. (1999) [7] and Virtanen et al. (2012) [9]. The higher prevalence of fractures in men likely reflects greater exposure to high-energy trauma, such as road traffic accidents, which accounted for 60% of injuries in our series. Falls on an outstretched hand caused 30% of injuries, a pattern commonly observed in trauma settings in developing countries.

Fracture distribution in our cohort showed that 83.3% involved the midshaft, while 16.7% involved the lateral third of the clavicle. This aligns with the known predominance of midshaft fractures, which are the most frequently displaced and surgically treated. Radiological outcomes were excellent, with 100% union achieved at a mean of 12.8 ± 2.3 weeks. These findings are comparable to those of Shen et al. [7], who reported a median union time of 10 weeks. Similarly, the Canadian Orthopaedic Trauma Society (COTS) trial demonstrated faster union with operative management (16.4 weeks) compared with nonoperative care (28.4 weeks) [10,16].

Functional outcomes were encouraging. At six months postoperatively, 80% of patients achieved “excellent” Constant-Murley Scores (≥90), 16.7% achieved “good,” and 3.3% achieved “fair,” with a mean score of 91.2 ± 6.4. These results align with Virtanen et al. (2012) [9], underscoring the importance of anatomical reduction and stable fixation for optimal shoulder function after ORIF. Complications were infrequent (16.7%), limited to superficial infections (6.7%) and hardware irritation (10%). No implant failures or nonunions occurred. These outcomes are similar to those reported by Zlowodzki et al. [18], who observed infection rates of 4-8% and hardware-related complaints in 10-15% of cases.

Randomized controlled trials and meta-analyses consistently support operative treatment for displaced midshaft fractures. Operative management is associated with lower nonunion rates, fewer symptomatic malunions, faster union, and improved early function. The COTS trial reported two nonunions in the operative group, compared with seven in the nonoperative group. Additionally, nine symptomatic malunions occurred in the nonoperative group, whereas none occurred in the surgical group [10]. Some systematic reviews suggest that, when nonunion is excluded, long-term functional differences may be minimal, emphasizing the need for careful patient selection [19].

Our findings support ORIF with pre-contoured LCPs as a safe and effective treatment for displaced midshaft and lateral-third clavicular fractures in adults. Younger, more active patients particularly benefit from an early return to function. Surgical decision-making should balance the advantages of anatomical reduction and early mobilization against risks, such as infection, hardware irritation, and potential implant removal. Treatment should be individualized, considering patient comorbidities, activity level, fracture displacement, shortening, comminution, and patient preference.

Strengths and limitations

Strengths of this study include its prospective design, standardized surgical technique, and use of a validated functional scoring system (Constant-Murley). All procedures were performed by experienced orthopedic surgeons, minimizing variability in technique. Limitations include the small sample size (n=30), which restricts statistical power and generalizability. The six-month follow-up allowed assessment of union and early functional recovery but was insufficient to evaluate long-term outcomes, such as late hardware failure, symptomatic hardware, or implant removal. The absence of a nonoperative control group also limits direct comparison between surgical and conservative management. Larger multicenter randomized studies in the Indian context are needed to address these gaps.

## Conclusions

Operative management of displaced clavicular fractures using a pre-contoured locking compression plate provides favorable functional and radiological outcomes, with reliable fracture union and a low complication rate. This technique provides stable fixation, restores clavicular alignment, and facilitates early mobilization, thereby improving shoulder function and overall recovery. When combined with meticulous surgical technique and a structured rehabilitation protocol, plate fixation allows a predictable return to pre-injury activity levels. Based on this prospective observational study, these findings support plate fixation as a safe and effective treatment for displaced clavicular fractures in adults, particularly for fracture patterns that may be associated with suboptimal outcomes if managed nonoperatively.
